# GALC Triggers Tumorigenicity of Colorectal Cancer via Senescent Fibroblasts

**DOI:** 10.3389/fonc.2020.00380

**Published:** 2020-04-07

**Authors:** Mengdi Yang, Zhiyuan Jiang, Guangyu Yao, Zhiyu Wang, Jing Sun, Huanlong Qin, Hui Zhao

**Affiliations:** ^1^Department of Internal Oncology, Shanghai Jiao Tong University Affiliated Sixth People's Hospital, Shanghai, China; ^2^Department of Gastrointestinal Surgery, Shanghai Tenth People's Hospital Affiliated With Tongji University, Shanghai, China

**Keywords:** senescent fibroblasts, tumorigenicity, colorectal cancer, galactosylceramidase, tumor microenvironment

## Abstract

Colorectal cancer (CRC)-associated senescent fibroblasts may play a crucial role in tumor progression, but the mechanism remains unclear. In order to solve this complicated problem, we randomly collected 16 patients with CRC, who had been treated with oxaliplatin and capecitabine (XELOX). Hematoxylin-eosin (HE) staining revealed that the tumor-stroma ratio (TSR) of CRC was affected by XELOX treatment. Immunohistochemistry (IHC) and senescence-associated β-galactosidase (SAβG) staining were used to verify a stable model of senescent fibroblasts. IHC analysis showed that high expression levels of galactosylceramidase (GALC) and significant senescence-associated β-galactosidase (SAβG) staining were associated with CRC patient survival. We observed that fibroblasts overexpressing GALC underwent cell cycle arrest. Changes in cell morphology and cell cycle characteristics were accompanied by the upregulation of the *p16, p21*, and *p53* gene, and the downregulation of *hTERT* expression. In a co-culture system, fibroblasts overexpressing GALC significantly increased the proliferation of CRC cells. Transmission electron microscopy (TEM) analysis confirmed that GALC overexpression fibroblasts co-cultured with CRC caused changes in CRC cell morphology. The aging fibroblast co-culture group (70%) had a higher migration ability. *In vivo* experiments and transcriptomics analysis were performed to verify the effect of senescent fibroblasts on tumor formation and to identify the potential mechanisms for the above results. We found that a high expression of ATF3 was related to good survival rates. However, a high expression of KIAA0907 was bad for survival rates (*p* < 0.05). The knockdown of ATF3 can promote cell proliferation, migration, and clonogenic assays, while downregulation of KIAA0907 inhibits cell proliferation, migration, and clonogenic assays. The results demonstrate that senescent fibroblasts with a high level of GALC regulated several aspects of the tumor growth process, including migration and invasion.

## Introduction

Colorectal cancer (CRC) remains one of the leading causes of mortality worldwide, and it is a severe threat to public health (1, 2). Treatment of CRC is a critical challenge, since many patients do not respond to therapy and those that do respond can develop drug resistance after most advanced treatment strategies that are provided in the clinics (3, 4). Previous studies have demonstrated that senescent fibroblasts are abundant and heterogeneous in the tumor microenvironment (TME), and they are closely associated with cancer progression and resistance to therapy (5, 6). As fibroblasts are the most abundant cell type in the tumor stroma, the deregulation of secreted paracrine factors from fibroblasts has been shown to influence the growth, invasion, and metastasis of cancer cells (7). While several mechanisms have been reported for the regulation of cancers by senescent fibroblasts (8–10), it is clear that additional mechanisms also contribute to the stromal regulation of cancers, and thus, additional studies are warranted.

Senescent fibroblasts are thought to be precursors to cancer-associated fibroblasts (CAFs) (11, 12). They share the ability to stimulate proliferation and invasive behavior (13, 14). Senescence was originally used as a model to study the aging of fibroblasts both *in vitro* and *in vivo* (15). However, senescent cells induced by traditional methods are difficult to obtain in large quantities. In cell culture experiments and in aging humans, senescent fibroblasts have been associated with increased β-galactosidase (SAβG) activity (16). Galactosylceramidase (GALC) is a lysosomal protein that hydrolyzes the galactose ester bonds of galactosylceramide, galactosylsphingosine, lactosylceramide, and monogalactosyldiglyceride (17). High levels of GALC expression can increase the expression of β-galactosidase. Thus, the cell senescence status can be assessed by detecting GALC expression. Metastatic CRC remains one of the most malignant human gastrointestinal carcinomas, with one of the worst 5-year prognoses (18, 19). While there is known to be an interaction between fibroblasts and CRC cells (20), the mechanisms are yet to be fully elucidated. Hence, this study aims to examine the effect of senescent fibroblasts on various CRC cell phenotypes.

## Materials and Methods

### Human Tissue Samples

The present study was reviewed and approved by the Ethics Committee of Shanghai Jiao Tong University Affiliated Sixth People's Hospital (2017-037). Written informed consent was obtained from all patients. The enrolling criteria included the following: (1) Colonoscopy diagnosed as CRC. (2) The preoperative imaging data were clearly T3-4N1-2M0, and the preoperative staging must have reached stage IIIB–IIIC (according to the American Cancer Society TNM staging standard) without distant metastasis. (3) Patients must be older than 18 years old and younger than 80 years old, and have a Kamofsky score of 70 or higher, with no history of tumor-related bleeding. (4) White blood cell count >4 × 10^9^/L, platelets >100 × 10^9^/L. The clearance rate of creatinine should be >60 ml/min, and there is sufficient liver function reserve: serum bilirubin <2.5 times the upper limit of normal, AS/ALT <2.5 times the upper limit of normal. (5) The patient had no previous bowel surgery and no history of radiotherapy and chemotherapy. The patient does not have any other malignant diseases. All patients received at least three cycles of the XELOX regimen with a 3-week course (oxaliplatin 130 mg/m^2^, day 1; capecitabine 1,250 mg/m^2^ twice daily, days 1–14). (6) Puncture samples from patients enrolled should be usable to perform clinical IHC and TSR analysis. (7) Patients should be available for follow-ups. Sixteen patients undergoing surgery after neoadjuvant chemotherapy at Shanghai Sixth People's Hospital and Shanghai Tenth People's Hospital between January 2010 and December 2012 were selected for this study. Biopsy specimens were collected from each patient before and after chemotherapy. The colonoscopy results before chemotherapy confirmed the diagnosis of CRC, and the computed tomography (CT) and magnetic resonance imaging (MRI) data of all patients were available. The characteristics of the patients included in this study are listed in Table 1.

**Table 1:** Basic information of clinical patients.

**Characteristics**			**Number**	**Percent (%)**
Gender	Man		9	56.25
	Woman		7	43.75
Tumor site				
	Colon			
		Ascending colon	4	25
		Sigmoid colon	3	18.75
	Rectum			
		High position (>10 cm)	3	18.75
		Median (7–10 cm)	2	12.5
		High position(<7 cm)	4	25
Surgical approach				
		Laparoscopic radical resection	10	62.5
		Dixon	3	18.75
		Miles	3	18.75
Pathology				
		Medium differentiated adenocarcinoma	5	31.25
		Low-grade adenocarcinoma	7	43.75
		Mucinous adenocarcinoma	4	25

### Cell Culture

Fibroblasts HFL1 (ATCC® CCL-153), HFF-1 (ATCC® SCRC-1041), CRC cell lines LoVo (ATCC® CCL-229), RKO (ATCC® CRL-2577), HCT116 (ATCC® CCL-247), HT-29 (ATCC® HTB-38), and virus-packaging 293T (ATCC® CRL-11268) cells were all purchased from the Institute of Biochemistry and Cell Biology, Chinese Academy of Science (Shanghai, China). The details of these cell lines can be obtained from the American Type Culture Collection (https://www.atcc.org/products). HFL1, HFF-1, LoVo, RKO, HCT116, HT-29, and virus-packaging 293T cells were cultured in Dulbecco's Modified Eagle Medium (DMEM; Invitrogen, Carlsbad, CA, USA), supplemented with 10% fetal bovine serum (FBS) and 1% penicillin/streptomycin, at 37°C under 5% CO_2_.

### Lentivirus Packaging

The transfection mixture was prepared according to the following ratio: 600 μl OPTI-MEM plus, 72 μl PEI, and 24 μg plasmid [PLVX-GFP-GALC, PLVX-GFP, shCK, shATF3 (shATF3-1: TGCTGTTGACAGTGAGCGCAAAGAGGCGACGAGAAAGAAATAGTGAAGCCACAGATGTATTTCTTTCTCGTCGCCTCTTTTTGCCTACTGCCTCGGA; shATF3-2:TGCTGTTGACAGTGAGCGCAAAGAGGCGACGAGAAAGAAATAGTGAAGCCACAGATGTATTTCTTTCTCGTCGCCTCTTTTTGCCTACTGCCTCGGA), shKIAA0907(shKIAA0907-1:TGCTGTTGACAGTGAGCGACTGGTGGTAGCTGAAGTAGAATAGTGAAGCCACAGATGTATTCTACTTCAGCTACCACCAGGTGCCTACTGCCTCGGA, shKIAA0907-2:TGCTGTTGACAGTGAGCGATAGATTTGTGAATCAGATTAATAGTGAAGCCACAGATGTATTAATCTGATTCACAAATCTAGTGCCTACTGCCTCGGA)], which included 12-μg target plasmid, 10.68-μg dR8.9, and 1.32-μg VSV-G. Then the mixture was allowed to stand for 10 min. Transfection occurred for 4–6 h, and the medium was then changed. The medium was collected at 48 and 72 h, respectively.

### SAβG Staining

A Senescence β-Galactosidase Staining Kit (Beyotime, Shanghai, China; C0602) was used for SAβG staining. The SAβG staining efficiency was calculated as the number of positively stained cells divided by the total number of cells in a single field of view. Fibroblasts with a staining rate greater than 10% were used for subsequent experiments.

### Cell Co-culture

CRC (LoVo, RKO, HCT116, HT29) cells were thawed and plated on 10-mm glass coverslips (Menzel Glaser; Braunschweig, Germany) in 24-well plates for co-culture experiments or directly onto Transwell chambers (24 wells, each with a 4 μm pore size polycarbonate membrane; Corning Incorporated, USA) for cell migration experiments. LoVo and RKO cells were separately added to a 24-well plate at 5 × 10^5^ cells per well. LV-GALC and LV-NC HFL1 cells were then seeded in the lower chamber of the transwell chamber at 2 × 10^5^ cells per well, with three replicate wells per group. Co-culture experiments were performed in duplicate and repeated on three independent occasions. Data from the three independent experiments were pooled.

### Animal Experiments

Experimental animals were ordered through the Animal Ethics Committee of Shanghai Jiao Tong University Affiliated Sixth People's Hospital, and all animal experiments were performed under a protocol approved by the Committee (2016-0137). Two million viable LV-GALC, LV-NC, and LoVo cells were injected subcutaneously into the following two groups of 6-week-old nude male mice: (A) LoVo and LV-GALC (*n* = 12); and (B) LoVo and LV-NC (*n* = 11). Mice were sacrificed when the tumor volume in control mice reached 1,200 mm^3^. Tumor volume (mm^3^) was calculated based on the formula for approximating the volume of a spheroid. The tumor volume is calculated as V = (Length × width^2^)/2.

### Tumor-Stroma Ratio

The tumor-stroma ratio (TSR) was first used for the early evaluation of colon cancer in 2007 (21). The percentage of each field of view occupied by tumor cells was then evaluated under a microscope at 200 × magnification, and the remaining area was considered as the percentage of stroma. At least two fields of view were selected for evaluation, and the highest percentage of stroma was used as the final value. TSR calculations were performed by at least two certified pathologists who were blind to the patients' information.

### Immunofluorescence

The immunofluorescence assay was performed using the primary antibodies against Ki67 (Abcam, #ab15580, 1:100). The samples were then incubated with 1:200 for 1 h, and then incubated with 4′,6-diamidino-2-phenylindole (DAPI) (Santa Cruz TM) for 15 min. The images were captured and analyzed using Leica TCS SP8.

### Immunohistochemical Studies

Paraffin-embedded sections were incubated with a primary antibody against ki-67 (Abcam, #ab15580, 1:100), GALC (Proteintech #21544-1-AP), p53 (CST #2527, 1:200), p21 (CST #2947, 1:100), and p16 (Abcam, #ab51243, 1:500), followed by incubation with a secondary biotinylated antibody (Kirkegaard & Perry Laboratories). The method used for immunohistochemistry has been described previously (22, 23).

### Cell Cycle Analysis

Cell cycle analysis of LV-NC-HFL1/HFF-1, LV-GALC-HFL1/HFF-1, and/or HFL1/HFF-1 co-cultured with CRC cell lines was performed using propidium iodide (PI) staining (Beyotime, Shanghai, China; C1062). The experiment was performed according to the instructions of the reagent. The cells (5 × 10^5^) were analyzed using a BD LSR Fortessa (BD Biosciences, USA), and the data were analyzed using the FlowJo software (TreeStar, USA).

### Quantitative Reverse-Transcriptase PCR Analysis

Triplicate samples of total RNA were transcribed into complementary DNA (cDNA) using AMV Reverse Transcriptase (Promega, Madison, Wisconsin, USA). qPCR was performed on the cDNA samples using gene-specific primers, the Maxima TM SYBR Green/ROX qPCR Master Mix (Fermentas, Glen Burnie, Maryland), and a 7300HT real-time PCR instrument (Applied Biosystems, Foster City, CA, USA). PCR results were evaluated by melting curve analysis and by confirming the expected PCR products on 2% (w/v) agarose gels. The following equation was used for the analysis: ΔC_T_ = C_Ttargetgene_ - C_T__Internalreferencegene_; the comparison between samples: ΔΔC_T_ = ΔC_T__Experimentalgroup−_ ΔC_T__Controlgroup_; foldchange = 2^−ΔΔ*CT*^. The sequences of all PCR primers are listed in Table 2.

**Table 2:** RT-PCR primers in this study.

**Genes**	**Sequences**
galc Forward	TATTTCCGAGGATACGAGTGGT
galc Reverse	CCAGTCGAAACCTTTTCCCAG
p16 Forward	GATCCAGGTGGGTAGAAGGTC
p16 Reverse	CCCCTGCAAACTTCGTCCT
p21 Forward	GGGGACCTAGAGCAACTTACT
p21 Reverse	CAGCGCAGTCCTTCCAAAT
tert Forward	GGCACGGCTTTTGTTCAGAT
tert Reverse	TCCGGGCATAGCTGGAGTAG
p53 Forward	AGCTTGATCGCCTCTATAAGGA
p53 Reverse	CCCTCAGCTCATTAACACGCT

### Western Blotting Analysis

Total cell lysates were prepared using a RIPA buffer. Equal amounts of protein were separated by electrophoresis, transferred onto polyvinylidene fluoride membranes, and incubated with primary antibodies against anti-p53 (CST #2527, 1:1,000), anti-p21 (CST #2947, 1:1,000), anti-p16 (Abcam, #ab51243, 1:1,000), and anti-GADPH (Abcam #8245, 1:2,000), and a Horseradish peroxidase-conjugated secondary antibody (Jackson ImmunoResearch, West Grove, PA, USA, 1:5,000) was used; blots were developed with the ECL Plus reagent (Millipore, Burlington, MA, USA).

### Transmission Electron Microscope Analysis

Samples were placed into 5-ml centrifuge tubes, fixed in glutaraldehyde for 1 h at room temperature, and then stored at 4°C for 4 h. After fixation, the samples were washed three times with 0.2 M phosphate buffer (pH 7.4) for 10 min each wash. The samples were then serially dehydrated in ethanol at concentrations of 30, 50, 75, 90, 95, and 100% for 10 min at each concentration. After desiccation in a drying oven for 12 h, the samples were fixed on the copper plates of the microscope for analysis by transmission electron microscopy (TEM).

### RNA-Seq

After 24 h of co-culture, as described above, the Transwell chamber was discarded and RNA was extracted from co-cultured LoVo cells in a 6-well plate for RNA-seq analysis. The samples were sent to Genechem (Shanghai, China) for RNA-seq library preparation. The library quality was assessed on the Agilent Bioanalyzer 2100 system. Three biological replicates were used for RNA-seq experiments. Sequencing libraries were generated using a NEBNext Ultra^TM^ RNA Library Prep Kit for Illumina (NEB, USA). The clustering of the index-coded samples was performed on a cBot Cluster Generation System using a TruSeq PE Cluster Kit v4-cBot-HS (Illumia), following the manufacturer's instructions.

### Transwell Assays

In total, 1 × 10^5^ cells were seeded into the upper Transwell chambers (Corning, NY, USA), and media with 10% FBS were added to the lower chamber. After incubation for 24 h, the chamber was fixed in methanol and then stained using crystal violet (Beyotime). Using a light microscope, at least five randomly selected fields were photographed, after which the counts were averaged. All experiments were performed in triplicate.

### Cell Count Kit-8

The cells were cultured in a 96-well plate for 0, 24, 48, 72, and 96 h. Thereafter, a Cell Count Kit-8 (CCK-8, Dojindo, Japan) with a medium volume of 10% was added into the wells and incubated for 2–4 h at 37°C. The absorbance (OD) of the solution was then measured using a microplate reader (Biorad, USA) at 450 nm. The experiments were carried out in sextuplicate.

### Colony Formation Assay

Cells were plated in a six-well plate and incubated at 37 °C for 2 weeks. Colonies were fixed with 4% phosphate-buffered formalin (pH 7.4) and stained with Giemsa for 15 min. Each experiment was performed in triplicate.

### Statistical Analysis

Data were analyzed by ANOVA, the chi-square test, or the two-tailed Student's *t*-test, the Fisher-exact test, and the Mann–Whitney *U*-test, as appropriate, using SPSS version 20.0 (SPSS Inc., Chicago, IL, USA). All data are presented as the mean ± SD, and ^*^p < 0.05, ^**^p < 0.01, ^***^p < 0.001 was considered to be statistically significant. All experiments were performed on three independent occasions.

## Results

### Analysis of Interstitial Cell Senescence and Related Pathological Parameters in Patients Undergoing Neoadjuvant Chemotherapy for Advanced CRC

**Figure 1:**
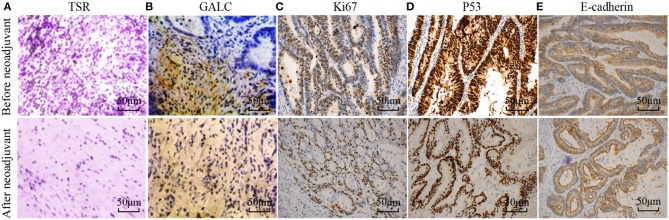
Disease-related indicators before and after neoadjuvant chemotherapy for colorectal cancer. **(A)** Hematoxylin-eosin (HE) staining showing the tumor-stroma ratio before and after neoadjuvant chemotherapy. Immunohistochemical staining of GALC **(B)**, Ki67 **(C)**, p53 **(D)**, and E-cadherin **(E)** was performed to explore the relationship between these markers and prognosis. *p* < 0.05 was considered statistically significant.

To understand the changes that occur in the tumor stroma in patients undergoing neoadjuvant chemotherapy for CRC, 16 patients with locally advanced CRC, who had been treated with oxaliplatin and capecitabine (XELOX), were randomly selected. HE staining was performed on tumor samples to determine TSR before and after (Figure 1A) neoadjuvant chemotherapy. The characteristics of the patients included in the study are listed in Table 1. We found that 37.50% of patients (*n* = 6) had increased TSR after chemotherapy; however, changes in total TSR had no significant effect on the prognosis of patients (*p* = 0.3) (Table 3). We also stained tumor tissue samples for GALC, Ki67, p53, and E-cadherin (Figures 1B–E) to explore the relationship between these markers and prognosis. The results showed that high levels of GALC expression were associated with a poor prognosis (*p* < 0.01; Table 4).

**Table 3:** The relationship between the clinical pathological factors and prognosis.

**TSR**	**State**		***p***
	**Survival (*n* = 11)**	**Died (*n* = 5)**	
Up	3	3	0.3
Down or not change	8	2	

**Table 4:** The relationship between the immunohistochemical markers and prognosis.

**Proteins**	**Expression**	**State**		***P***
		**Survival (*n* = 11)**	**Died (*n* = 5)**	
GALC	H	1	5	<0.01**
	L	10	0	
Ki67	H	7	4	0.6
	L	4	1	
p53	H	5	2	1
	L	6	3	
E-cadherin	H	4	2	1
	L	7	3	

### Generation of Senescent Fibroblasts Through GALC Overexpression

**Figure 2:**
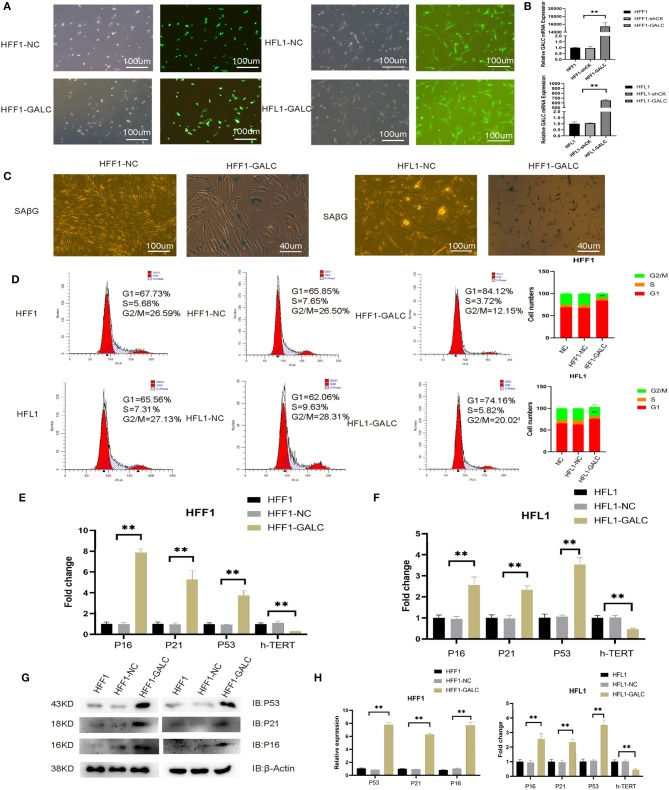
Generation of senescent fibroblasts, and the cell cycle and senescence-associated markers in LV-GALC HFF1/HFL1 fibroblasts cells. **(A)** Construction of the LV-GALC HFF1/HFL1 fibroblasts cells. **(B)** The expression of GALC in normal HFF1/HFL1 cells (NC), LV-NC HFF1/HFL1 fibroblasts, and LV-GALC HFF1/HFL1 fibroblast cells. **(C)** β-galactosidase staining of LV-NC HFF1/HFL1 fibroblasts and LV-GALC HFF1/HFL1 fibroblast cells. **(D)** Cell cycle analysis of NC, LV-NC HFF1/HFL1 fibroblasts, and LV-GALC HFF1/HFL1 fibroblast cells. **(E,F)** qRT-PCR analysis of *hTERT, p16, p21*, and *p53* expression in NC, LV-NC HFF1/HFL1 fibroblasts, and LV-GALC HFF1/HFL1 fibroblast cells. **(G)** The expression of p53, p21, and p16 proteins in NC, LV-NC, and LV-GALC HFF1/HFL1 cells. **(H)** Quantitative analysis of the expression of p16, p53, and p21 proteins in NC, LV-NC, and LV-GALC HFF1/HFL1 cells.

To explore the mechanism for the association between high GALC expression fibroblasts and poor prognosis in CRC patients, we transfected fibroblast cells with PLVX-GFP (LV-NC) or PLVX-GFP-GALC (LV-GALC) vectors (Figure 2A). We then determined the expression of GALC in LV-NC, LV-GALC, and un-transfected HFF1/HFL1 cells (NC) using qRT-PCR (*p* < 0.05, Figure 2B). These results demonstrated the successful overexpression of GALC in LV-GALC HFL1/HFF-1 fibroblasts. We also observed senescent features (24), including increased SAβG staining (Figure 2C) and morphological changes to larger, more flattened, and more irregularly shaped cells in LV-GALC HFF1/HFL1 cells, not observed in LV-NC HFF-1/HFL1 cells.

### GALC-Overexpressing Senescence Fibroblast Cells

Relative to LV-NC and NC fibroblast cells, there was a significant increase in the number of LV-GALC HFF1/HFL1 fibroblasts in the G0/G1 phase and a significant decrease in the number of cells in the G2/M phase (Figure 2D). We further examined the cell cycle profile and the expression of additional protein markers associated with senescence, including *p16, p21*, and *p53*. LV-GALC HFF1/ HFL1 fibroblast cells had elevated *p16, p21*, and *p53* mRNA levels (Figures 2E,F, *p* < 0.05), while LV-GALC cells had lower (*p* < 0.05) levels of *hTERT* mRNA. The protein expression of P16, P21, and P53 was found to be higher in LV-GALC HFF1/HFL1 fibroblast cells than in LV-NC and NC fibroblast cells (Figures 2G,H). Overall, our results demonstrated that GALC overexpression led to the senescence of HFF1/HFL1 fibroblast cells.

### Impact of Senescent Fibroblasts on CRC Cells

**Figure 3:**
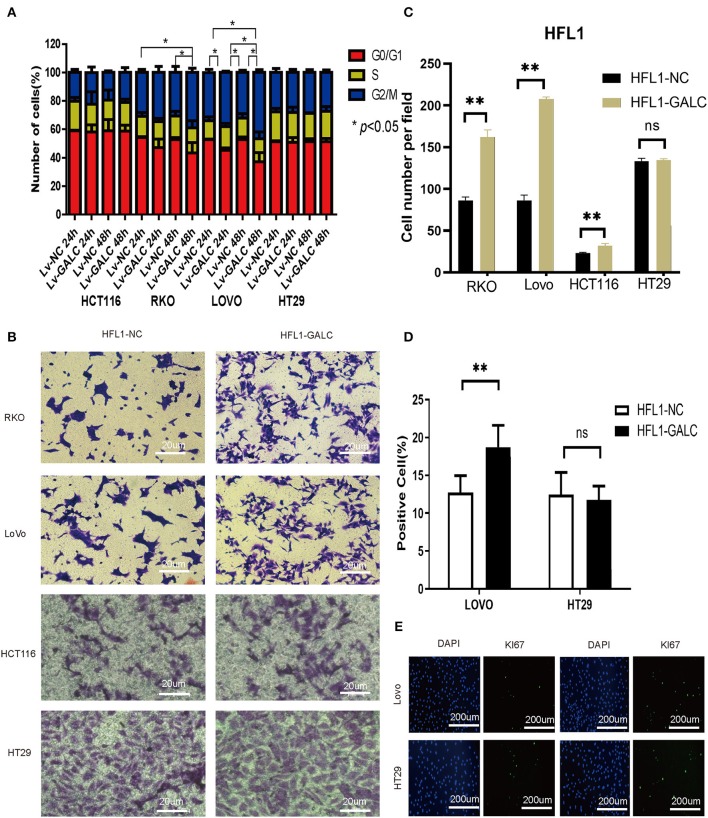
*In vitro* impact of senescent fibroblasts on CRC cells. **(A)** After co-culture with LV-GALC fibroblasts cells, the proportion of G0/G1 phase LoVo cells shown at 24 and 48 h (*p* < 0.05) and the proportion of G0/G1 phase RKO cells shown at 48 h (*p* < 0.05). **(B,C)** Migration ability in a Transwell migration assay for RKO, LoVo, HCT116, and HT29 cells co-cultured with LV-GALC fibroblast cells and control cells (*p* < 0.05). **(D,E)** Ki-67 staining of LoVo cells co-cultured with LV-GALC fibroblast cells (*p* < 0.05). *p* < 0.05 was considered statistically significant.

We sought to determine the effects of LV-GALC fibroblast cells on several aspects of tumor regulation in co-culture models with CRC cells. Through cell cycle profile analysis, we observed a decreased percentage of RKO and HCT116 cells in the G0/G1 phase, but an increased percentage in the G2/M phase, when they were co-cultured with LV-GALC fibroblast cells. The most significant effect was seen in RKO cells at 48 h (*p* < 0.05). A similar phenomenon was observed in LoVo cells after 24 and 48 h of co-culture (*p* < 0.05); however, there was no enrichment of HT29 cells in the G2/M phase (Figure 3A). In the Transwell migration assays, RKO and LoVo cells co-cultured with LV-GALC HFL1 fibroblast cells had significantly enhanced cell mobility (*p* < 0.05). The mobility of HT29 cells co-cultured with LV-GALC HFL1 fibroblast cells was not different from their mobility when co-cultured with LV-NC HFL1 fibroblast cells (Figures 3B,C). We also examined the proliferation indices represented by Ki67 expression in LoVo and HT29 cells. The proliferation indices for LoVo cells when co-cultured with LV-NC and LV-GALC fibroblast cells were 12.72 ± 2.26% and 18.71 ± 2.88%, respectively (*p* < 0.05). However, no apparent changes were seen in the proliferation of HT29 cells (Figures 3D,E).

### Changes in the Structure of CRC Cells Co-cultured With Senescent Fibroblasts

**Figure 4:**
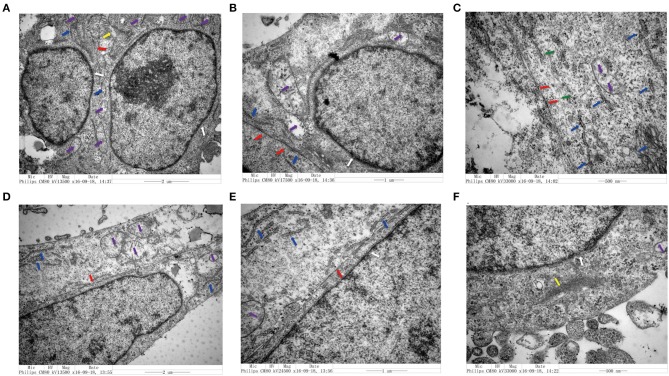
Transmission electron microscopy analysis of colorectal cell lines in the presence and absence of senescent fibroblasts. **(A–C)** Structural characteristics of the LoVo cells that co-cultured with LV-GALC fibroblasts and **(D–F)** LoVo cells that co-cultured with LV-NC fibroblasts. Nuclear membrane (white arrow), mitochondria (purple arrow), visible increase in rough endoplasmic reticulum ribosomes (blue arrow), tight connection between cells (red arrow), microfilament (yellow), and microtube (green).

We utilized TEM to examine the structure of CRC cells co-cultured with senescent LV-GALC HFL1 fibroblasts (Figures 4A–C) or LV-NC HFL1 fibroblasts (Figures 4D–F). We observed 10 cells in each group and found that seven of the aging fibroblast co-culture group (70%) contained cell morphological changes, compared with only two in the control group (20%). The aging fibroblast co-culture group (70%) had a higher migration ability: large nuclear heteromorphism, nuclear chromatin accumulation, increased mitochondria (Purple arrow), visible increase in rough endoplasmic reticulum ribosomes (Blue arrow), fewer tight connection between cells (Red Arrow), microfilament (Yellow), microtube (Green), changes in cell polarity, with more elongated protrusions and foot processes, and an increase in the number of extracellular microvilli, which are not easily observed in attached and centrifuged cells. We observed that senescent fibroblasts induced notable morphological changes in the cancer cell cytoskeletal structure, with an increased number of microfilament structures. These changes may contribute to the mobility of the cancer cells and potentially enhance their metastatic capacity.

### *In vivo* Impacts of Senescent Fibroblasts on CRC

**Figure 5:**
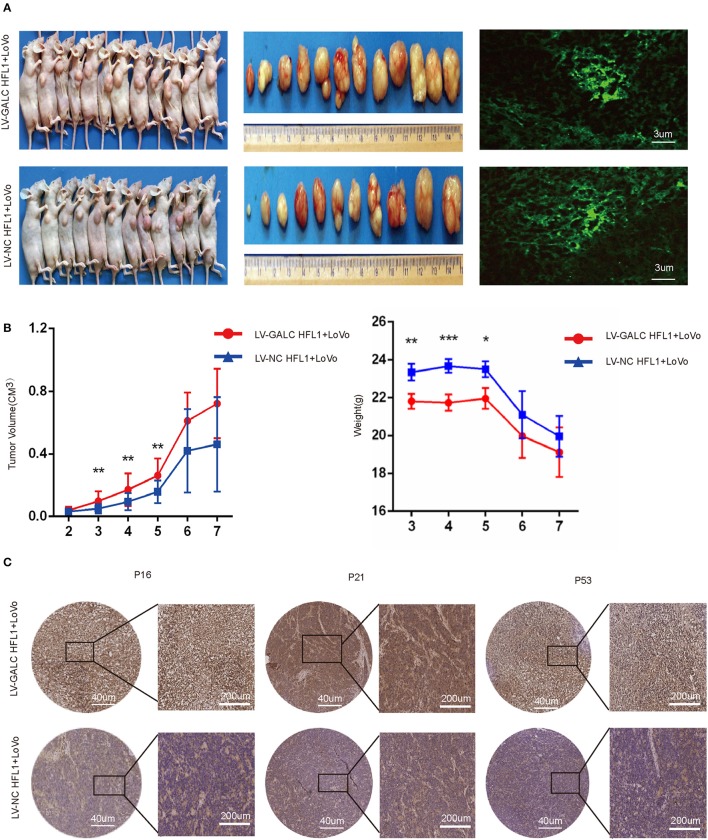
*In vivo* impact of senescent fibroblasts on cancer cells. **(A)** Group A (LoVo and LV-GALC fibroblast cells) and Group B (LoVo and LV-NC fibroblast cells) mice, tumors sizes, and green fluorescence staining were shown. Tumor volume **(B)** and weight data for the two groups of mice. *p* < 0.05 was considered statistically significant. **(C)** Group A (LoVo and LV-GALC fibroblast cells) and group B (LoVo and LV-NC fibroblast cells) mice tumors were stained with P16, P21, and P53 using IHC.

To further investigate the *in vivo* effects of senescent fibroblasts on CRC, we also implanted xenografts of LV-GALC HFL1 fibroblasts co-cultured LoVo cells into nude mice. Seven days after the subcutaneous injection of cancer cells, 12 group A mice (LV-GALC HFL1 fibroblasts and LoVo cells) and 11 group B mice (LV-NC HFL1 fibroblasts and LoVo cells) demonstrated tumor formation (Figure 5A). Group A mice showed a statistically significant increase in tumor volume compared to group B mice. In the first 2 weeks, there was no significant difference in the tumor volume between the tumors of group A mice and those of group B. After the third week, in concordance with tumor growth, mice in group A demonstrated significantly greater weight loss compared to mice in group B (Figure 5B). When senescent fibroblast and tumor cells were first inoculated in mice, senile fibroblasts promoted tumor growth. However, the phenotype of senile fibroblasts was lost as the cells were inoculated for a longer period. Finally, we investigated the expression of p16, p21, and p53 (Figure 5C) in groups A and B mice. We found that P16, P21, and P53 were more highly expressed in group A mice (LV-GALC fibroblasts and LoVo cells) than group B mice (LV-NC fibroblasts and LoVo cells). Overall, our results suggested that exogenous senescent fibroblasts likely contribute to the grafting and growth of tumor cells.

### Transcriptomics and Biochemical Analysis Revealed the Putative Mechanism Underlying the Impact of Senescent Fibroblasts on CRC

**Figure 6:**
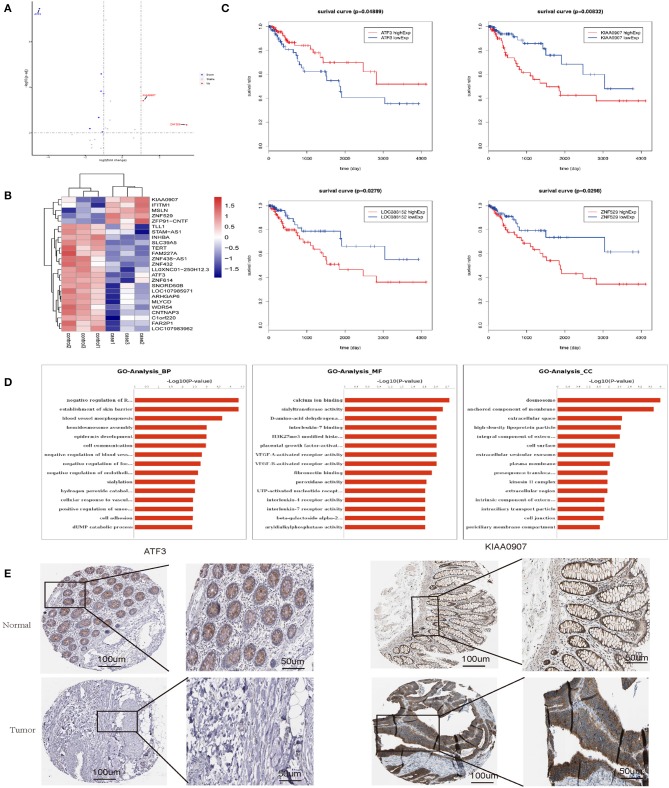
Transcriptomics and biochemical analysis revealed the putative mechanism for the impact of senescent fibroblasts on CRC. **(A)** Volcano map and **(B)** heat map analyses were performed to identify genes that were differentially expressed between LV-GALC and LV-NC fibroblast cells co-cultured with LoVo cells. **(C)** Survival analysis of ATF3, KIAA0907, LOC388152, and ZNF529 using datasets from the TCGA database. **(D)** Gene ontology pathway enrichment analysis suggested that co-culture LV-GALC fibroblasts with LoVo led to the activation of several pathways. **(E)** The expression of ATF3 and KIAA0907 in CRC and normal colon tissue by IHC [data from The Human Protein Atlas (www.proteinatlas.org)]. *p* < 0.05 was considered statistically significant.

To elucidate the mechanism by which senescent fibroblasts promote the tumor properties of CRC cells, we performed transcriptomics and biochemical analysis of CRC co-cultured with LV-GALC and LV-NC fibroblast cells. First, we constructed Volcano maps (Figure 6A) and Heat maps (Figure 6B) to identify the genes differentially expressed between these two groups. Next, we examined the expression of several genes using the TCGA database and found that low levels of ATF3 expression and high levels of KIAA0907, LOC388152, and ZNF529 expression contributed to tumorigenicity. Furthermore, based on TCGA follow-up and statistical analyses, we found that low levels of ATF3 expression and high levels of KIAA0907, LOC388152, and ZNF529 expression were significantly associated with reduced survival (*p* < 0.05, Figure 6C). Gene ontology (GO) pathway enrichment analysis [biological process (BP), molecular function (MF), and cellular component (CC)] suggested that co-culturing with senescent fibroblasts led to the activation of several pathways associated with tumor cell survival and metastasis (Figure 6D). We also analyzed the expression of ATF3 and KIAA0907 in CRC using The Human Protein Atlas (www.proteinatlas.org). Compared with normal tissues, ATF3 is weakly expressed in CRC, while KIAA0907 is highly expressed in CRC (Figure 6E).

### ATF3 and KIAA0907 Are Closely Related to Tumorigenesis and Metastasis

**Figure 7:**
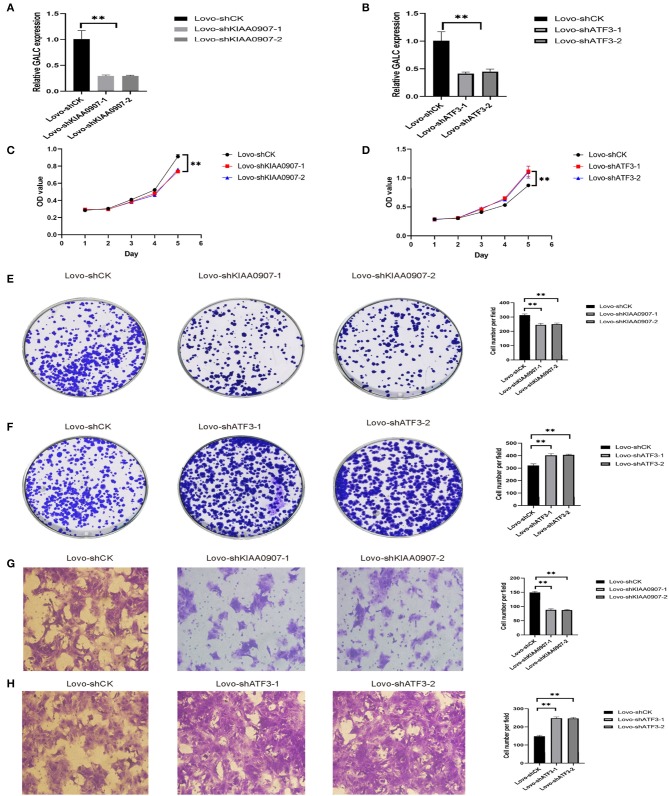
ATF3 and KIAA0907 are closely related to tumorigenesis and metastasis. Knockdown efficiency of KIAA0907 **(A)** and ATF3 **(B)**. CCK8 assays showed the cell proliferation after Knockdown KIAA0907 **(C)** and ATF3 **(D)**. Colony formation assays showed the proliferation after Knockdown KIAA0907 **(E)** and ATF3 **(F)**. Transwell assay revealed the cell migration after Knockdown KIAA0907 **(G)** and ATF3 **(H)**.

In order to verify the transcriptome results, we knocked down KIAA0907 (Figure 7A), which was highly expressed, and ATF3 (Figure 7B), which was weakly expressed. We then conducted the CCK8 (Figures 7C,D) and colony formation (Figures 7E,F) assay to explore the influence of ATF3 and KIAA0907 on CRC. We found that the knockdown of ATF3 promoted cell proliferation and the knockdown of KIAA0907 inhibited cell proliferation. The Transwell assay found that the knockdown of KIAA0907 (Figure 7G) inhibited cell migration, and the deregulation of ATF3 (Figure 7H) promoted it. These results revealed that LV-GALC fibroblast cells co-cultured with CRC upregulates oncogenes and downregulates tumor suppressor genes, thereby affecting tumor progression.

## Discussion

CRC is the fourth most common cancer diagnosed in adults and the second leading cause of death from cancer in the United States (25). Neoadjuvant systemic chemotherapy is advocated by current treatment guidelines (26). However, not all neoadjuvant chemotherapies are effective for all patients. A better understanding of the biology of CRC is imperative for the development of more effective therapeutic approaches (27).

Cellular senescence is a stable state of proliferative arrest that provides a barrier to malignant transformation and contributes to the antitumor activity of certain chemotherapies (28, 29). Senescent fibroblasts are already highly resistant to chemotherapy (30). Specifically, senescent stromal cells have been shown to play a role in carcinogenesis (31). Senescent fibroblasts can stimulate cancer cell proliferation and invasion (32). The results from this study demonstrated that tumors treated with chemotherapy were enriched in these stromal cells and this, in turn, worsened patient outcomes. These findings led us to subsequently examine the effect of senescent fibroblasts on the regulation of several phenotypes that are key to CRC tumorigenesis by GALC.

First, we established a stable model of senescent fibroblasts and we then used co-culture experiments to examine the effects of LV-GALC fibroblast effects on several tumor phenotypes relevant to CRC biology. The overexpression of GALC in HFL1/HFF-1 fibroblasts cells resulted in positive SAβG staining and the morphological changes to larger, more flattened, and more irregularly shaped cells that closely resembled senescent cells compared to LV-NC HFF1/HFL1 fibroblasts. We found that the percentage of LV-GALC cells in the G0/G1 phase was significantly higher than the percentage of control cells in the G0/G1 phase, whereas the percentage of LV-GALC cells in the G2/M phase was significantly lower, suggesting that cells have undergone cell cycle arrest. Notably, p53/P21/P16 is a vital signal axis that can induce cell senescence (33). We further identified LV-GALC senescent fibroblasts with higher G0/G1 cell cycle characteristics, which were accompanied by the upregulation of p16, p21, and p53 not only at the gene level but also in proteins. In co-culture experiments, LV-GALC fibroblast cells significantly increased the proliferation of LoVo cells and, expectedly, reduced the number of LoVo cells in the G0/G1 phase, while increasing those in the G2/M phase. *In vivo* experiments assessing subcutaneous tumor formation in mice showed that both tumor volume and tumor weight were greater in group A than in group B. In the early period when LV-GALC fibroblasts possess senescent properties (*p* < 0.05), P16, P21, and P53 were more highly expressed in group A mice (LV-GALC fibroblasts and LoVo cells) than group B mice (LV-NC fibroblasts and LoVo cells). This indicates that senescent interstitial fibroblasts can increase the tumorigenic ability of human CRC cells *in vivo*.

Understanding the transcriptome is essential for understanding development and disease processes (34, 35). In the present study, we performed RNA-seq experiments to further analyze the effects of co-culture on cell function and tumorigenesis. Furthermore, transcriptomics and biochemical analysis of CRC cells showed that there are many genes that are differentially expressed between co-cultures of LV-GALC senescent fibroblasts and LV-NC fibroblast cells. GO pathway enrichment analysis suggested that co-culturing CRC cells with senescent fibroblasts led to the activation of several pathways associated with tumor cell survival and metastasis. Cellular Component Ontology (CC) revealed that the co-culture group had more desmosome components. This is consistent with our TEM results, which further illustrated that CRC cell co-cultured with LV-GALC senescent fibroblasts had an increased number of microfilament structures, which may contribute to the mobility of cancer cells and potentially enhance metastatic capacity. ATF3 and KIAA0907 potentially contribute to tumorigenicity, and these factors were significantly associated with survival in the TCGA. We further explored the roles of ATF3 and KIAA0907 in CRC cells. We found that the knockdown of ATF3 promoted cell proliferation, migration, and clonogenic formation, while the deregulation of KIAA0907 inhibited it, which may explain the above results that senescent fibroblasts and CAFs share the ability to stimulate proliferation and invasive behavior and also indicate the potential mechanisms. Taken together, these results showed that senescent fibroblasts regulate the tumorigenicity of CRC cells and play important roles in tumor biology.

Herein, we demonstrated that senescent fibroblasts regulated several aspects of the survival and metastasis of CRC. Targeting these processes may improve the efficacy of clinical treatment. New therapeutic strategies should be developed based on our understanding of the regulatory roles of the TME in CRC.

## Data Availability Statement

Publicly available datasets were analyzed in this study, these can be found in the NCBI Gene Expression Omnibus (https://www.ncbi.nlm.nih.gov/geo/) (GSE145662).

## Ethics Statement

The studies involving human participants were reviewed and approved by The present study was reviewed and approved by the Ethics Committee of Shanghai Jiao Tong University Affiliated Sixth People's Hospital (2017-037). The patients/participants provided their written informed consent to participate in this study. The animal study was reviewed and approved by the Animal Ethics Committee of Shanghai Jiao Tong University Affiliated Sixth People's Hospital and all animal experiments were performed under a protocol approved by the Committee (2016-0137).

## Author Contributions

MY: data curation, project administration, management, coordination responsibility for the research activity planning and execution and writing. ZJ: methodology, application of statistical, mathematical, and computational. GY: collected clinical samples and investigation. ZW: validation experiments and other research outputs. JS: provision of study materials, patients, laboratory samples, and instrumentation tools. HQ and HZ: formulation of overarching research goals and aims, supervision.

### Conflict of Interest

The authors declare that the research was conducted in the absence of any commercial or financial relationships that could be construed as a potential conflict of interest.

## References

[1] Bray F Ferlay J Soerjomataram I Siegel RL Torre LA Jemal A. Global cancer statistics 2018: GLOBOCAN estimates of incidence and mortality worldwide for 36 cancers in 185 countries. CA Cancer J Clin. (2018) 68:394–424. 10.3322/caac.21492 30207593

[2] Siegel RL Miller KD Jemal A Cancer statistics, 2018. CA Cancer J Clin. (2018) 68:7–30. 10.3322/caac.21442 29313949

[3] Siravegna G Mussolin B Buscarino M Corti G Cassingena A Crisafulli G Clonal evolution and resistance to EGFR blockade in the blood of colorectal cancer patients. Nat Med. (2015) 21:795–801. 10.1038/nm.3870 26030179 PMC4868598

[4] Punt CJ Koopman M Vermeulen L. From tumour heterogeneity to advances in precision treatment of colorectal cancer. Nat Rev Clin Oncol. (2017) 14:235. 10.1038/nrclinonc.2016.171 27922044

[5] Alspach E Flanagan KC Luo X Ruhland MK Huang H Pazolli E. p38MAPK plays a crucial role in stromal-mediated tumorigenesis. Cancer Discov. (2014) 4:716–29. 10.1158/2159-8290.CD-13-0743 24670723 PMC4049323

[6] Schubeler D. Function and information content of DNA methylation. Nature. (2015) 517:321–6. 10.1038/nature14192 25592537

[7] Bhowmick NA Neilson EG Moses HL. Stromal fibroblasts in cancer initiation and progression. Nature. (2004) 432:332–337. 10.1038/nature03096 15549095 PMC3050735

[8] Kim E Rebecca V Fedorenko IV Messina JL Mathew R Maria-Engler SS. Senescent fibroblasts in melanoma initiation and progression: an integrated theoretical, experimental, and clinical approach. Cancer Res. (2013) 73:6874–85. 10.1158/0008-5472.CAN-13-1720 24080279 PMC3926439

[9] Coppe JP Desprez PY Krtolica A Campisi J. The senescence-associated secretory phenotype: the dark side of tumor suppression. Annu Rev Pathol. (2010) 5:99–118. 10.1146/annurev-pathol-121808-102144 20078217 PMC4166495

[10] Wang H Tian L Goldstein A Liu J Lo HC Sheng K. Bone-in-culture array as a platform to model early-stage bone metastases and discover anti-metastasis therapies. Nat Commun. (2017) 8:15045. 10.1038/ncomms15045 28429794 PMC5413944

[11] Ohtani N Hara E. Roles and mechanisms of cellular senescence in regulation of tissue homeostasis. Cancer Sci. (2013) 104:525–30. 10.1111/cas.12118 23360516 PMC7657106

[12] Jayatilaka H Tyle P Chen JJ Kwak M Ju J Kim HJ. Synergistic IL-6 and IL-8 paracrine signalling pathway infers a strategy to inhibit tumour cell migration. Nat Commun. (2017) 8:15584. 10.1038/ncomms15584 28548090 PMC5458548

[13] Gorgoulis V Adams PD Alimonti A Bennett DC Bischof O Bishop C. Cellular senescence: defining a path forward. Cell. (2019) 179:813–27. 10.1016/j.cell.2019.10.005 31675495

[14] Taddei ML Cavallini L Comito G Giannoni E Folini M Marini A. Senescent stroma promotes prostate cancer progression: the role of miR-210. Mol Oncol. (2014) 8:1729–46. 10.1016/j.molonc.2014.07.009 25091736 PMC5528604

[15] Campisi J Robert L. Cell senescence: role in aging and age-related diseases. Interdiscip Top Gerontol. (2014) 39:45–61. 10.1159/000358899 24862014 PMC4211612

[16] Dimri GP Lee X Basile G Acosta M Scott G Roskelley C. A biomarker that identifies senescent human cells in culture and in aging skin *in vivo*. Proc Natl Acad Sci USA. (1995) 92:9363–7. 10.1073/pnas.92.20.9363 7568133 PMC40985

[17] Visigalli I Ungari S Martino S Park H Cesani M Gentner B. The galactocerebrosidase enzyme contributes to the maintenance of a functional hematopoietic stem cell niche. Blood. (2010) 116:1857–66. 10.1182/blood-2009-12-256461 20511539 PMC3173985

[18] Loupakis F Cremolini C Masi G Lonardi S Zagonel V Salvatore L. Initial therapy with FOLFOXIRI and bevacizumab for metastatic colorectal cancer. N Engl J Med. (2014) 371:1609–18. 10.1056/NEJMoa1403108 25337750

[19] Grothey A Van Cutsem E Sobrero A Siena S Falcone A Ychou M. Regorafenib monotherapy for previously treated metastatic colorectal cancer (CORRECT): an international, multicentre, randomised, placebo-controlled, phase 3 trial. Lancet. (2013) 381:303–12. 10.1016/S0140-6736(12)61900-X 23177514

[20] Calon A Lonardo E Berenguer-Llergo A Espinet E Hernando-Momblona X Iglesias M. Stromal gene expression defines poor-prognosis subtypes in colorectal cancer. Nat Genet. (2015) 47:320–9. 10.1038/ng.3225 25706628

[21] Mesker WE Junggeburt JM Szuhai K de Heer P Morreau H Tanke HJ. The carcinoma-stromal ratio of colon carcinoma is an independent factor for survival compared to lymph node status and tumor stage. Cell Oncol. (2007) 29:387–98. 10.1155/2007/175276 17726261 PMC4617992

[22] Yang M Sun Y Sun J Wang Z Zhou Y Yao G. Differentially expressed and survival-related proteins of lung adenocarcinoma with bone metastasis. Cancer Med. (2018) 7:1081–92. 10.1002/cam4.1363 29522283 PMC5911611

[23] Fromowitz FB Viola MV Chao S Oravez S Mishriki Y Finkel G. ras p21 expression in the progression of breast cancer. Hum Pathol. (1987) 18:1268–75. 10.1016/S0046-8177(87)80412-4 3315956

[24] Gu K Xu Y Li H Guo Z Zhu S Zhu S. Real-time tracking and *in vivo* visualization of beta-galactosidase activity in colorectal tumor with a ratiometric near-infrared fluorescent probe. J Am Chem Soc. (2016) 138:5334–40. 10.1021/jacs.6b01705 27054782

[25] Wolf AMD Fontham ETH Church TR Flowers CR Guerra CE LaMonte SJ. Colorectal cancer screening for average-risk adults: 2018 guideline update from the American Cancer Society. CA Cancer J Clin. (2018) 68:250–81. 10.3322/caac.21457 29846947

[26] Siriwardena AK Mason JM Mullamitha S Hancock HC Jegatheeswaran S. Management of colorectal cancer presenting with synchronous liver metastases. Nat Rev. Clin Oncol. (2014) 11:446–59. 10.1038/nrclinonc.2014.90 24889770

[27] Lu J Ye X Fan F Xia L Bhattacharya R Bellister S. Endothelial cells promote the colorectal cancer stem cell phenotype through a soluble form of Jagged-1. Cancer Cell. (2013) 23:171–85. 10.1016/j.ccr.2012.12.021 23375636 PMC3574187

[28] Narita M Narita M Krizhanovsky V Nunez S Chicas A Hearn SA. A novel role for high-mobility group a proteins in cellular senescence and heterochromatin formation. Cell. (2006) 126:503–14. 10.1016/j.cell.2006.05.052 16901784

[29] De Castro J Garcia R Garrido P Isla D Massuti B Blanca B. Therapeutic potential of denosumab in patients with lung cancer: beyond prevention of skeletal complications. Clin Lung Cancer. (2015) 16:431–46. 10.1016/j.cllc.2015.06.004 26264596

[30] Fane M Weeraratna AT. How the ageing microenvironment influences tumour progression. Nat Rev Cancer. (2020) 20:89–106. 10.1038/s41568-019-0222-9 31836838 PMC7377404

[31] Agrawal K Das V Taborska N Gursky J Dzubak P Hajduch M. Differential regulation of methylation-regulating enzymes by senescent stromal cells drives colorectal cancer cell response to DNA-demethylating Epi-drugs. Stem Cells Int. (2018) 2018:6013728. 10.1155/2018/6013728 30158986 PMC6109465

[32] Krtolica A Parrinello S Lockett S Desprez PY Campisi J. Senescent fibroblasts promote epithelial cell growth and tumorigenesis: a link between cancer and aging. Proc Natl Acad Sci USA. (2001) 98:12072–7. 10.1073/pnas.211053698 11593017 PMC59769

[33] Yang F Yi M Liu Y Wang Q Hu Y Deng H. Glutaredoxin-1 silencing induces cell senescence via p53/p21/p16 signaling axis. J Proteome Res. (2018) 17:1091–100. 10.1021/acs.jproteome.7b00761 29356545

[34] Wang Z Gerstein M Snyder M. RNA-Seq: a revolutionary tool for transcriptomics. Nat Rev Genet. (2009) 10:57–63. 10.1038/nrg2484 19015660 PMC2949280

[35] Dijkstra KK Voabil P Schumacher TN Voest EE. Genomics- and transcriptomics-based patient selection for cancer treatment with immune checkpoint inhibitors: a review. JAMA Oncol. (2016) 2:1490–5. 10.1001/jamaoncol.2016.2214 27491050

